# The Effectiveness of Osseodensification Drilling versus the Conventional Surgical Technique on Implant Stability: A Clinical Trial

**DOI:** 10.3390/jcm13102912

**Published:** 2024-05-15

**Authors:** João Fontes Pereira, Rosana Costa, Miguel Nunes Vasques, Marta Relvas, Ana Cristina Braga, Filomena Salazar, Marco Infante da Câmara

**Affiliations:** 1 Department of Medicine and Oral Surgery, University Institute of Health Sciences (IUCS-CESPU), 4585-116 Gandra, Portugal; joao.pereira@iucs.cespu.pt (J.F.P.); rosana.costa@iucs.cespu.pt (R.C.); miguel.vasques@iucs.cespu.pt (M.N.V.); marta.relvas@iucs.cespu.pt (M.R.); filomena.salazar@iucs.cespu.pt (F.S.); 2 Oral Pathology and Rehabilitation Research Unit (UNIPRO), University Institute of Health Sciences (IUCS-CESPU), 4585-116 Gandra, Portugal; 3 Algoritmi Centre, School of Engineering, University of Minho, 4800-058 Guimarães, Portugal; acb@dps.uminho.pt

**Keywords:** osseodensification, low bone density, implant stability, osseointegration, resonance frequency analysis, insertion torque

## Abstract

**Background/Objective:** To ensure that implants are able to support prosthetic rehabilitation, a stable and functional union between the bone and the implant surface is crucial to its stability and success. To increase bone volume and density and excel bone-implant contact, a novel drilling method, called osseodensification (OD), was performed. To assess the effectiveness of the osseodensification drilling protocol versus the conventional surgical technique on implant stability. **Methods:** Bone Level Tapered Straumann implants were placed side-by-side with both OD and subtractive conventional drilling (SD) in 90 patients from CESPU—Famalicão clinical unit. IT was measured using a manual torque wrench, and the Implant stability quotient (ISQ) value was registered using the Osstell^®^ IDX. **Results:** According to the multifactorial ANOVA, there were statistically significant differences in the mean IT values due to the arch only (F(1.270) = 4.702, *p*-value = 0.031 < 0.05). Regarding the length of the implant, there were statistically significant differences in the mean IT in the OD group (*p* = 0.041), with significantly lower mean IT values for the Regular implants compared to the Long. With respect to the arch, the analyses of the overall ISQ values showed an upward trend in both groups in the maxilla and mandible. High levels of IT also showed high ISQ values, which represent good indicators of primary stability. **Conclusions:** OD does not have a negative influence on osseointegration compared to conventional subtractive osteotomy.

## 1. Introduction

The placement of dental implants to restore the oral cavity has been incorporated into daily dental practice as a dental treatment alternative since Branemark PI et al. [1] revolutionized the total and partial rehabilitation of edentulous individuals. To ensure that implants are able to support prosthetic rehabilitation, a stable and functional union between the bone and the implant surface is crucial to its stability and success [2,3,4].

An established primary stability, which is characterized as sufficient contact between the implant and bone at their interface upon instrumentation and subsequent implant placement, is needed for successful osseointegration. Strong primary stability is therefore linked to increased osseointegration [5,6]. In dental implants, primary stability is a crucial factor for successful osseointegration, and the surgical procedure and bone density are important factors in achieving this stability. The dimensions of the osteotomy, the implant design, and the amount of bone strain imposed determine the degree of primary stability [2,3,4,7,8].

Insertion torque (IT), resonance frequency analysis (RFA), and the patient’s bone density are also related to the success of the implant’s primary stability. Thus, a high insertion torque significantly increases primary stability compared to an implant inserted with low insertion torque values [7]. As seen in the human jaw, for example, low-density bone can lead to poor contact between the bone and the implant, which can negatively affect primary stability and secondary stability. Thus, an adequate volume of bone in the implant site preparation is crucial to ensure osseointegration and long-term implant stability [2,9].

Secondary stability is also necessary for osseointegration, which is established over time due to bone remodeling around the implant during the healing period [2,10].

In order to increase the bone volume and density, which are essential for achieving bio-mechanically stable bone–implant contact, Huwais S. introduced a novel drilling method in 2013, called osseodensification (OD), which has revolutionized the world of implantology [9]. Conventional drilling protocols have employed a clockwise (subtractive) cutting technique with a positive angle of inclination, which has resulted in the absence of bone debris in the osteotomy. However, the non-cutting (additive) drilling technique, osseodensification, has been shown to compact the walls of the osteotomy site through lateral displacement of the bone, increasing primary stability. Moreover, the compacting of residual bone remains, which function as nucleating surfaces for osteoblasts after the implant, acts as an autograft that promotes osteointegration [10,11]. Thus, OD promotes an increase in peri-implant bone density, autologous bone compaction, plastic bone deformation, and an increase in the primary stability of the implant due to the viscoelastic characteristics of the alveolar bone, using a specific set of Densah^®^ Burs (Versah^®^ LLC, Jackson, MI, USA) in a counterclockwise direction at a speed of 800 to 1500 rpm [12,13]. This benefit can be crucial for decreasing implant micromovement during osseointegration and achieving high success rates in low-density bone. Another advantage is the reduction in the size of the osteotomies when the drills are removed, called the spring-back effect, due to the viscoelastic part of the bone deformation. In addition, it promotes higher insertion torques and, thus, enables immediate loading in comparison with conventional subtractive drilling techniques [9]. For this purpose, insertion torque (IT) and resonance frequency analyses were measured at three different times: (i) surgical phase of implant placement (T1); (ii) 6 months after implant placement (T2); and (iii) 1-year follow-up (T3).

## 2. Materials and Methods

### 2.1. Study Design

This study was designed as a clinical trial study according to CONSORT guidelines [14]. The interventions were approved by the Ethical Committee of the of the University Institute of Health Sciences (reference: 02/CE-IUCS/2019), and conducted in compliance with the provisions of the declaration of Helsinki. The study was registered in the ISRCTN registry (registration number ISRCTN15797074).

### 2.2. Patient Selection

All patients underwent a preliminary assessment that included a careful analysis of their medical and dental histories and a detailed clinical examination. Patients were thoroughly informed, by means of oral and written explanations, about the purpose and procedures of the study, and informed consent was obtained from all participants.

For inclusion, participants must be at least eighteen years old, have healed edentulous sites on the posterior maxillae region with at least 3 months of postextraction period; need to receive at least two dental implants; and have sufficient residual bone volume for implant placement without the need for bone augmentation where the minimum ridge height and width should be ≥8 and ≥6 mm, respectively. The exclusion criteria were: alcoholism, drug abuse, diabetes, heart disease, bleeding disorders, weakened immune systems, radiation exposure, past or ongoing use of steroids or bisphosphonates, and previous bone regenerative or augmentation procedures.

From 6 February to 10 March 2019, 120 patients from the CESPU—Famalicão clinical unit were screened from this patient pool, 90 of whom met the study’s inclusion criteria and were selected to participate.

In order to perform a comparison between osseodensification (OD) and subtractive conventional drilling (SD), the implants were placed side by side or contralateral, with both techniques to establish a comparison in RFA and torque values. In some patients, two implants were placed, but in other patients, they had between 3 and 4 implants.

Two independent examiners (J.F.P/M.I.C) were used to demonstrate intra- and inter-examiner reliability, measurements of the clinical parameters of implant primary stability were repeated in 50% of the sample.

### 2.3. Pre-Operative Radiographic Planning

The pre-operative radiographic examination: panoramic X-ray (and a cone beam computed tomography (CBCT, New Tom^®^ Go 3D, CEFLA S.C., Imola (BO) Italy) were used for initial participants screening. CBCT was crucial in order to provide a guide for assessing the condition of the Schneiderian membrane, ostium patency, presence of antral septa, and other pathologies that may influence the alveolar bone and the degree of sinus pneumatization and thickness of Schneiderian membrane.

### 2.4. Presurgical Phase

All patients underwent scaling 8 days prior to implant surgery. During this phase, preoperative instructions were given:

- To eat a light diet, avoiding fatty, fried, laxative and fermentable foods (milk, cheese, bananas) on the day of surgery.
- Not to wear jewelry or make-up, in the case of women.
- Avoid smoking in the 72 h before and 30 days after surgery, to avoid anesthetic and surgical complications, as well as contributing to better tissue healing.
- Not to take medication based on acetylsalicylic acid (aspirin) in the 4 days before surgery.
- Start antibiotic therapy 48 h before surgery (875 mg of amoxicillin and 125 mg of clavulanic acid) twice daily for 8 days.

### 2.5. Surgical Phase

#### 2.5.1. Implant Design and Surface Characteristics

Bone Level Tapered (BLT) Straumann^®^ implants (Basel, Switzerland) with a CrossFit^®^ connection (Basel, Switzerland) 3.3.mm diameter (Narrow connection—NC) or 4.1/4.8 mm diameter (Regular connection—RC) were used. These implants feature a tapered, self-cutting design with a 0.8 mm thread pitch, and are designed for excellent primary stability. BLT implants are available in lengths of short (8 mm), Regular (10 mm, 12 mm, 14 mm) and Long (16 mm, 18 mm). The implant surface SLA^®^ (Basel, Switzerland), Sandblasted, large grit, acid-etched is a type of surface treatment that creates surface roughness with the goal of enhancing osseointegration through greater bone-to-implant contact (BIC).

#### 2.5.2. Conventional Protocol

Patients were prepared and long-acting local anesthesia was administered (4% articaine with 1:100.000 adrenaline). 

A mid crestal incision was made and a full thickness mucoperiosteal flap was raised. The anterior region of the edentulous area was prepared using subtractive conventional drilling, according to the Straumann guidelines of RPM values. Independent of the type of implant diameter selected for the site (Ø 3.3 mm, 4.1 mm or 4.8 mm), the narrower drill (pilot drill Ø 2.2 mm, 800 rpm) was used until reached the desired depth under abundant saline irrigation. All drills were used in clockwise rotation. The drilling sequence is shown in Table 1, Table 2 and Table 3. After using the drill sequence, the BLT implant delivered in the implant bed. 

#### 2.5.3. Osseodensification Protocol

The posterior region of the edentulous area was prepared using the osseodensification procedure to test what has already been described in the literature, which is that this technique is especially used in situations of low-density bone (type III/IV) (which is typically found in the posterior region of the maxilla/mandible) in order to increase bone volume, percentage of bone to implant contact and, subsequentially, the primary stability of the implant.

Drilling was carried out to the desired depth using the pilot drill (clockwise drilling speed from 800 to 1500 rpm) with abundant saline irrigation. Depending on the implant diameter selected (Ø 3.3,4.1 or 4.8 mm), the narrower Densah^®^ Bur (Bur 1 for each implant diameter- Table 4) was used in a counterclockwise direction (800 to 1500 rpm) with a pumping motion until reaching the desired depth under abundant irrigation. All drills were used in counterclockwise rotation. The drilling sequence is shown in Table 4.

After using the drill sequence, the osteotomy received a threaded Sandblast large grit acid-etched (SLA) implant. In some cases, we finished placing the implant with a ratchet wrench, when the drill motor that drives the implant into place has reached the maximum placement torque.

**Figure 1:**
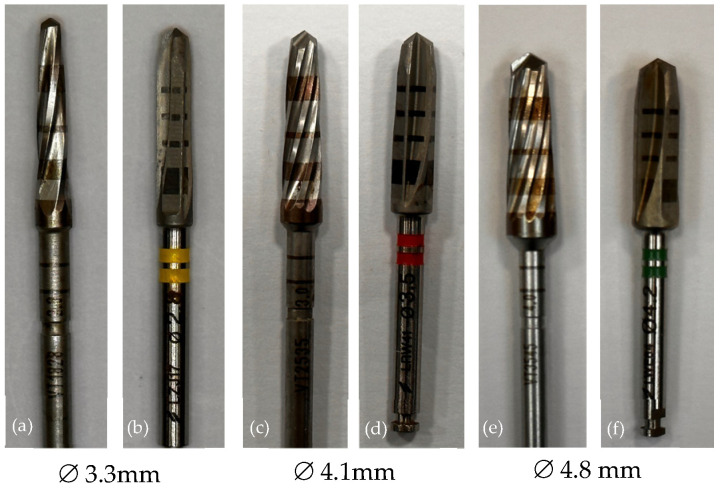
The configuration of the final drill used for each group (SD and OD). (**a**) VT1828; (**b**) Ø 2.8 BLT Drill; (**c**) VT2535; (**d**) Ø 3.5 BLT Drill; (**e**) VT3545; (**f**) Ø 4.2 BLT Drill.

Figure 1 shows the final drill used for each group (SD and OD) and implant diameters (Ø 3.3 mm, Ø 4.1 mm, Ø 4.8 mm).

#### 2.5.4. Evaluation of Implant Stability Parameters

Immediately after implant placement, the IT was measured (T1) using a manual torque wrench (Straumann^®^, Basel, Switzerland), and the Implant stability quotient (ISQ) value was registered as the average of the buccal, lingual, mesial and distal readings using the Osstell^®^ IDX (Osstell, W&H, Gothenburg, Sweden). IT and ISQ values were measured in all implants placed with the SD and OD technique.

A cover screw was placed in all implants. 

Afterwards, the surgical site was closed with several interrupted sutures using a monofilament suture (Nylon, Resorba^®^ 4.0, Nuremberg, Germany).

**Figure 2:**
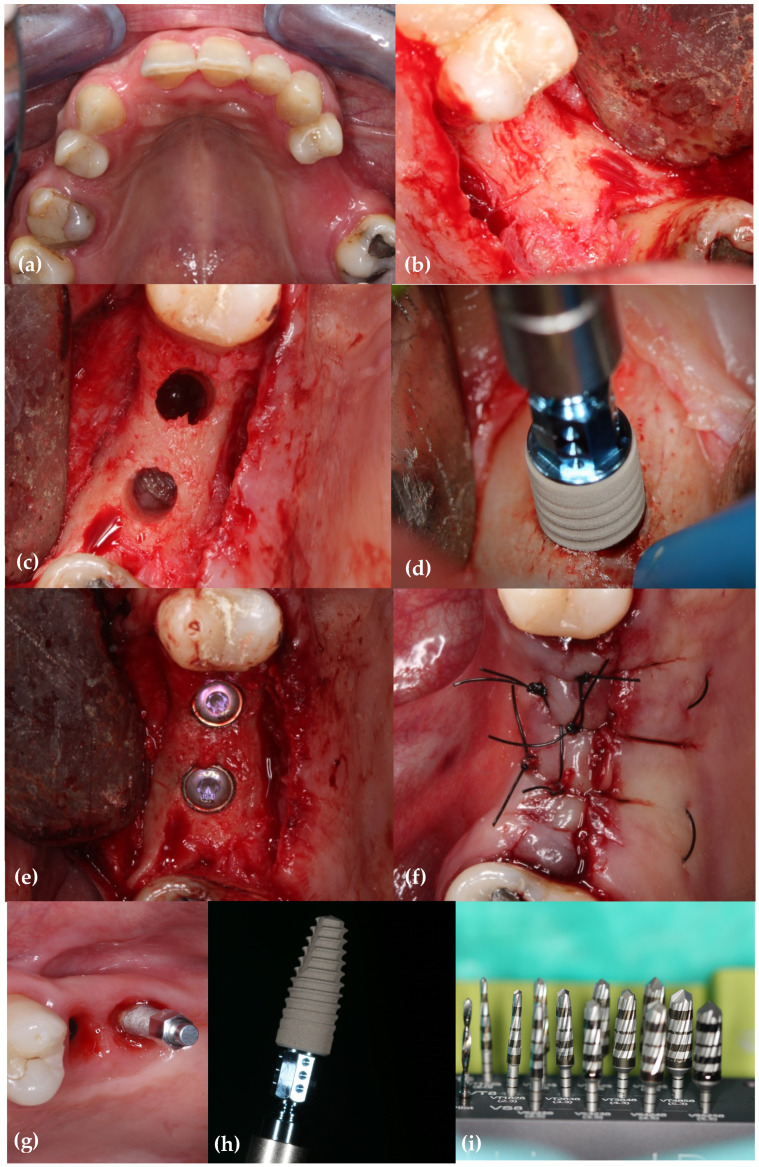
A visual representation of the surgery. (**a**) The initial case; (**b**) full thickness mucoperiosteal flap; (**c**) osteotomies; (**d**) implant placement (Ø 4.1 mm BLT); (**e**) a view of the cover screws; (**f**) the interrupted sutures using a monofilament suture (Nylon, Resorba^®^4.0); (**g**) SmartPeg placement for ISQ reading; (**h**) tapered implant design; (**i**) Densah Bur kit.

Figure 2 illustrates OD osteotomies.

### 2.6. Postoperative Instructions

Postoperative instructions were given of some important actions to avoid increased edema (swelling), pain, bleeding and infections:

- To lie down for the first three days after surgery to stabilize the blood clot, as this is a critical period for a good post-operative result without complications.
- To continue the antibiotic therapy and to take Naproxen (500 mg) twice daily for a 3 day period.
- Paracetamol 1 g 3 times a day for pain control management.
- To use 0.12% chlorhexidine gluconate mouthwash (Bexident^®^ Post Isdin, Barcelona, Spain) thrice daily for two weeks to reduce plaque formation.
- To apply ice to their faces in the first 6 to 8 h after surgery in order to significantly reduce facial edema, while also improving pain control and reducing local vascularization, thus preventing bleeding.
- To prepare a liquid/pasty and cold diet for 8 days.
- To bite on a piece of sterile gauze for 30 min to promote hemostasis.
- Not to spit, which could cause negative pressure in the mouth and dislodge the clot. Drinking liquids through straws is also contraindicated.
- Avoid vigorous mouthwashes.
- Not to smoke during the entire osseointegration process (especially during the first two weeks). Nicotine destroys vitamin C, which is essential for tissue regeneration, delaying the repair of the surgical wound.
- To refrain from physical activity or heavy lifting for three days after surgery.

After the post-operative indications were made, the patients were scheduled to have the sutures removed ten days after surgery.

### 2.7. Healing Abutment—6 Months

After six months of healing, the survival of the implants was verified, and the secondary stability was measured though ISQ values (T2), and an appropriate healing abutment was inserted considering the emergence profile and gingival height. Subsequently, the patients were scheduled for digital implant impressions with the 3Shape^®^ scanner (Copenhagen, Denmark), and final ceramic crowns were manufactured.

### 2.8. One Year Follow-Up

Removal of screw-retained zirconia crowns and ISQ values were recorded (T3) using the Osstell^®^ IDX.

### 2.9. Statistical Analysis

Descriptive statistics were calculated for each variable, including mean values and the corresponding 95% confidence interval (CI). The quantitative variables were assessed for normality using the Kolmogorov–Smirnov test and Normal probability graphical methods (QQ-plot), and the fit to the normal distribution was verified. The homogeneity of variances was assessed using Levene’s test.

The factorial ANOVA model test and multiple comparison tests were carried out to compare torque/torque values. Repeated measures analysis of variance and the respective Tukey tests for multiple comparisons were used to analyze the ISQ data. Pearson’s correlation test was applied to investigate the relationship between IT and immediate ISQ values for all the variables studied. 

All analyses were carried out using IBM^®^ Statistical Program for Social Sciences (SPSS^®^) Statistics software, version 29.0 for Windows, with a significance level of 5%.

## 3. Results

Of the 120 patients screened at the CESPU—Famalicão clinical unit, only 90 met the study’s inclusion criteria and were selected to participate.

**Figure 3:**
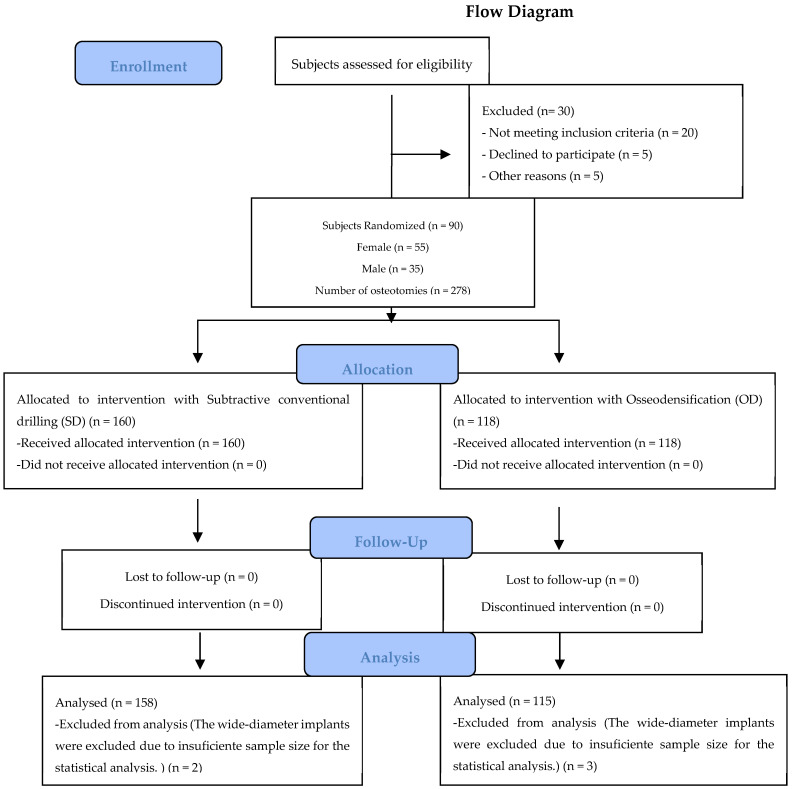
CONSORT flow chart.

Figure 3 illustrates the design of the study in the form of a CONSORT diagram. 

As shown in Table 5, the total sample consists of 90 individuals, 55 of whom are female (61.1%) and the remaining 35 male (38.9%). The limits of the 95% confidence intervals are also shown.

Table 6 shows the data characterizing the sample in terms of age by gender and overall.

Table 7 shows the results of the characterization of the sample in terms of the characteristics of the individuals assessed, as well as the respective limits (Lower Control Limit (LCL) and Upper Control Limit (UCL)) of the 95% confidence intervals (CI).

Table 8 shows the sample characterization data regarding the implant for each surgical technique and as a whole.

To assess whether there are differences in IT, a multifactorial ANOVA was carried out, and it was found that there are statistically significant differences in the mean IT values due to the arch only (F(1.270) = 4.702, *p*-value = 0.031 < 0.05).

**Figure 4:**
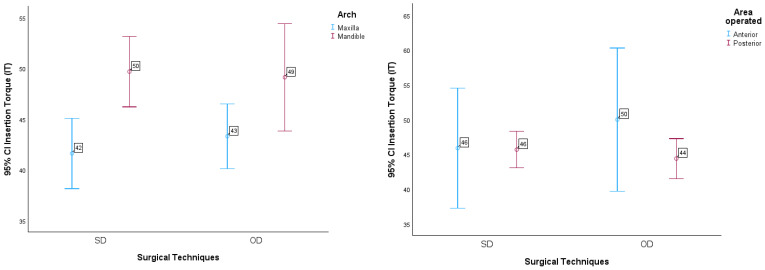
95% CI insertion torque in relation to surgical technique as a function of arch (maxilla and mandible) and area operated on (anterior and posterior).

The results can be seen in the graphs in Figure 4.

Another multifactorial ANOVA procedure was carried out, and statistically significant differences were found in mean IT values due to the effects of implant length (F(2.261) = 3.243, *p*-value = 0.041 < 0.05), and due to the effects of the interaction between technique used and implant diameter (F(1.261) = 4.538, *p*-value = 0.041 < 0.05), in the sense that the mean IT value with the SD technique for the Narrow implant is significantly lower when compared to the Regular.

The multiple comparison tests showed that the differences in the mean IT values with length are significantly lower for the Regular implants when compared to the Long implants (*p* = 0.011 < 0.05).

**Figure 5:**
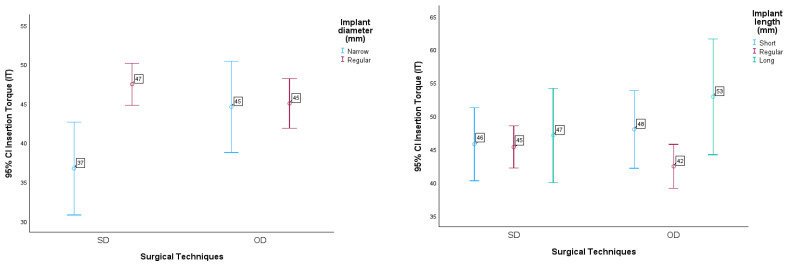
95% CI insertion torque in relation to surgical technique as a function of implant diameter (Narrow and Regular) and implant length (Short, Regular, Long).

These results are illustrated in the graphs in Figure 5.

To evaluate the effect of the different factors (surgical technique, arch and area operated) in relation to ISQ over time, a repeated measures ANOVA (three times) was performed.

**Figure 6:**
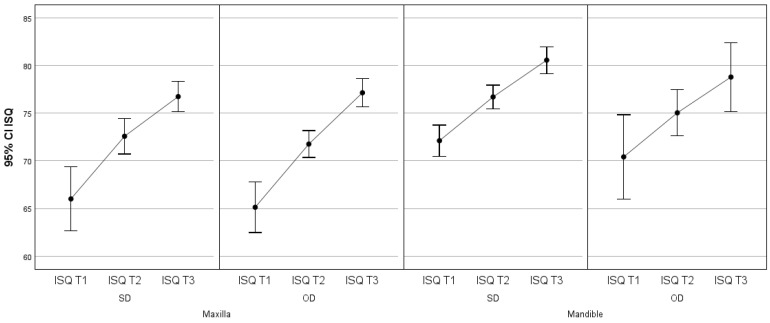
95% CI implant stability quotient in relation to surgical technique (SD and OD) at three different times (T1, T2 AND T3) in relation to arch (maxilla and mandible).

**Figure 7:**
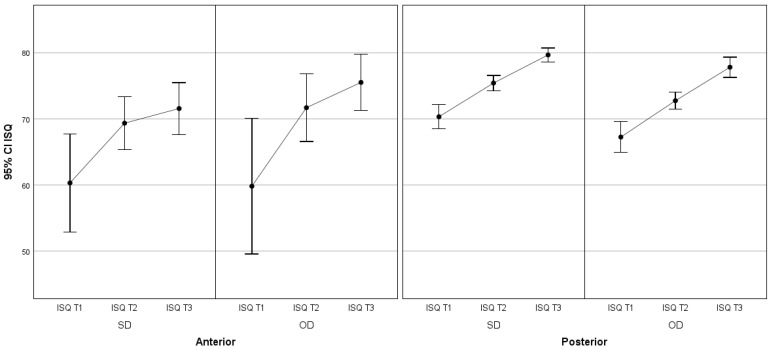
95% CI implant stability quotient in relation to surgical technique (SD and OD) at three different times (T1, T2 AND T3) in relation to area operated (anterior and posterior).

These results are illustrated in the graphs in Figure 6 and Figure 7.

Once the assumption of sphericity was tested using the Mauchly test (*p*-value < 0.05), the sphericity of the data was rejected. As the value of the epsilon estimate was less than 0.75, the Greenhouse–Geisser correction was used to interpret the results for intra-subject effects.

**Figure 8:**
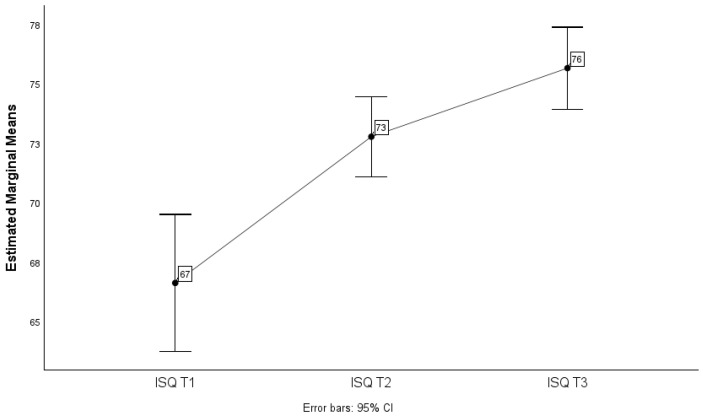
Distribution of mean ISQ values over time and respective 95% confidence intervals.

In this way, it was found that there are statistically significant differences in the average ISQ values in the different periods considered, i.e., there is significant variation in the average ISQ value over time, in the sense that it increases significantly over time (Figure 8). Statistically significant differences by multiple comparison tests (*p* < 0.05) were detected between all pairs (T1–T2, T1–T3 and T2–T3).

**Figure 9:**
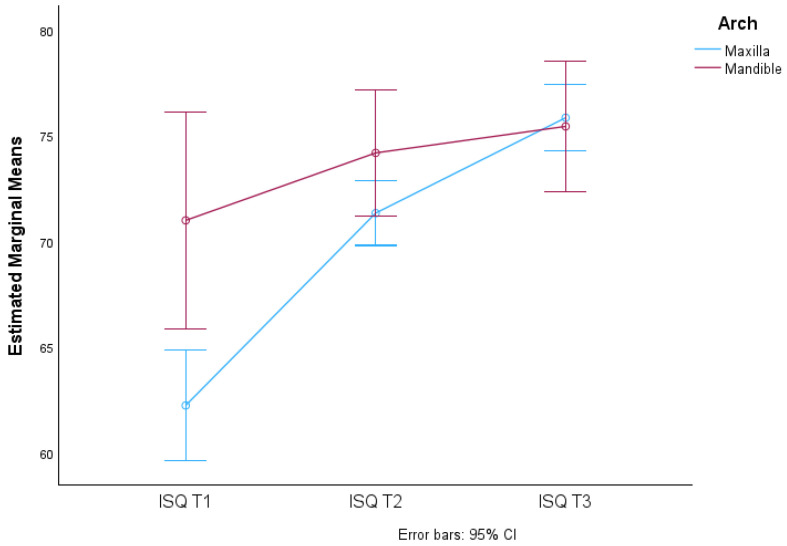
Distribution of mean ISQ values over time according to arch and respective 95% confidence intervals.

There were significant differences in the mean ISQ values due to the interaction of time and arch (F(1.438; 388.165) = 6.620, *p*-value < 0.05), which means that the means of the groups (maxilla and mandible) vary differently over the three times considered (T1, T2 and T3), i.e., the mean ISQ over time is not the same for the arches considered. This is reflected in the non-parallel lines in the graph in Figure 9.

**Figure 10:**
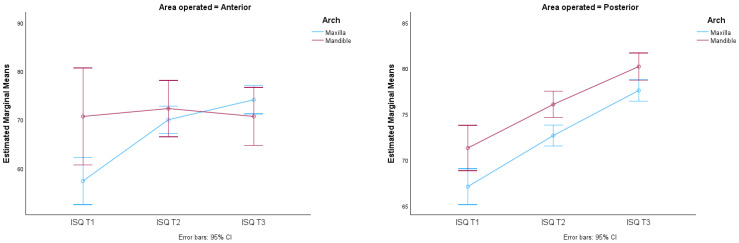
Distribution of mean ISQ values over time according to arch and area with respective 95% confidence intervals.

There were significant differences in the average ISQ values due to the interaction of time, arch and area (F(1.438; 388.165) = 4.553, *p*-value < 0.05), which means that the averages of the groups (maxilla and mandible) vary differently depending on the area of operation (posterior or anterior) in the three times considered, i.e., the average ISQ over time is not the same for the arch and area of operation considered. This is illustrated by the different behavior of the graphs in Figure 10.

As in the previous situation, to evaluate the effect of the different factors (surgical technique, diameter, and length) in relation to the ISQ over time, an ANOVA with repeated measures (three times) was carried out.

Once the assumption of sphericity was tested using the Mauchly test (*p*-value < 0.05), the sphericity of the data was rejected. As the value of the epsilon estimate is less than 0.75, the Greenhouse–Geisser correction will be used to interpret the results for intra-subject effects.

As with the previous results, there were statistically significant differences in the mean ISQ values in the different periods considered; statistically significant differences by multiple comparison tests (*p* < 0.05) were detected between all pairs (T1–T2, T1–T3 and T2–T3).

**Figure 11:**
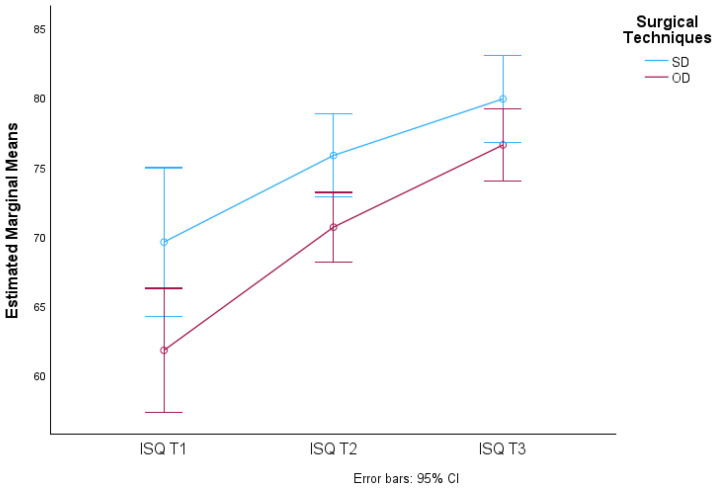
Distribution of mean ISQ values over time according to surgical procedure and respective 95% confidence intervals.

There were no significant differences in the average ISQ values due to the interaction of time and technique used (F(1.438; 375.336) = 0.163, *p*-value > 0.05), meaning that the ISQ averages over time in the groups (SD and OD) did not vary. This is reflected in the almost overlapping lines in the graph in Figure 11.

**Figure 12:**
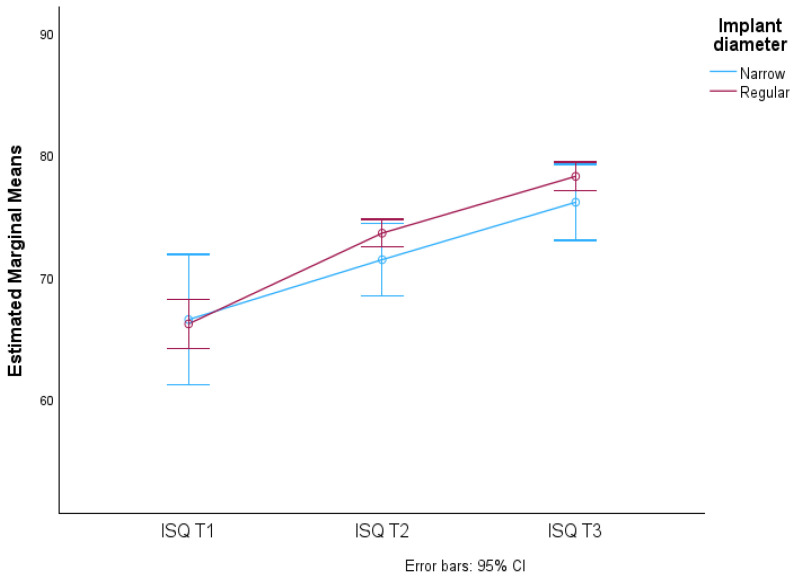
Distribution of mean ISQ values over time according to implant diameter and respective 95% confidence intervals.

No significant differences were found in the average ISQ values due to the interaction of time and technique used (F(1.438; 375.336) = 0.685, *p*-value > 0.05), meaning that the ISQ averages over time in the diameters considered (Narrow and Regular) do not vary. This is reflected in the almost overlapping lines in the graph in Figure 12.

**Figure 13:**
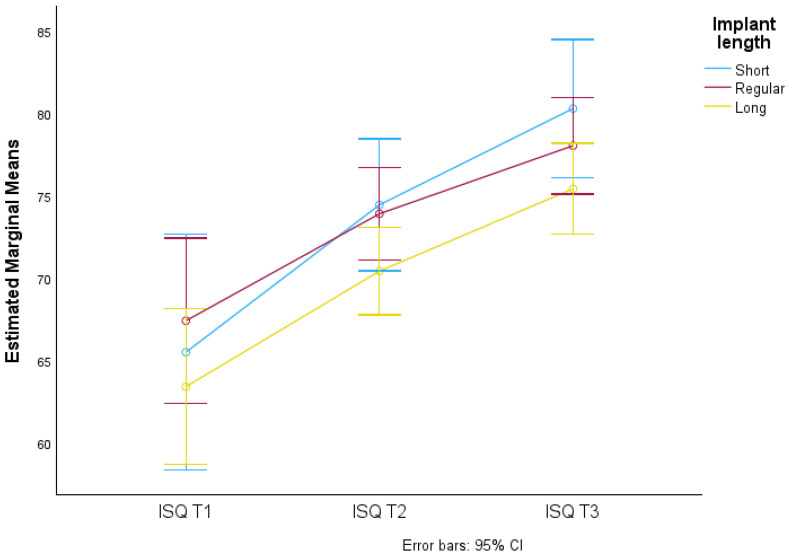
Distribution of mean ISQ values over time according to implant length and respective 95% confidence intervals.

**Figure 14:**
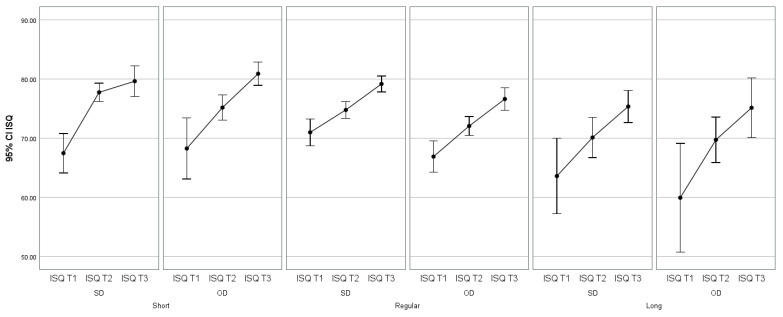
95% CI Implant stability quotient in relation to surgical technique (SD and OD) at three different times (T1, T2 and T3) in relation to implant length (Short, Regular, and Long).

There were no significant differences in the average ISQ values due to the interaction of time and technique used (F(2.876; 375.336) = 1.014, *p*-value > 0.05), meaning that the ISQ averages over time in the lengths considered (Short, Regular, and Long) do not vary. This is reflected in the lines and confidence limits, which are practically superimposed on the graph in Figure 13 and Figure 14.

Table 9 shows the correlation beteen th IT and ISQ T1 values and the variables under study.

## 4. Discussion

Osseointegration and primary implant stability are objectives of critical importance because their impediments often lead to implant failure [15]. Implant primary stability is a crucial component of osseointegration, and is correlated with bone density, surgical drilling technique, implant surface texture, and geometry [4,16,17]. Besides primary stability, it is important for the implant to obtain secondary stability, which is achieved after bone production and maturation on the implant body [16]. For this reason, the application of tests to assess the primary and secondary stability of the implant has become extremely important in implant dentistry. These tests include determining the insertion torque (IT) and resonance frequency analysis (RFA) [17].

Previous studies focused on the analysis of OD effects on implant placement, the present study evaluated the OD drilling effects on healing in three different stages T1, T2 and T3 with different diameters and lengths placed in anterior and posterior regions of the maxilla and in the mandible. To assess the implant stability, insertion torque measurements and resonance frequency analysis were carried out.

The IT, initially developed by Johansson and Strid, is applied with a torque wrench, and is the measure of the frictional resistance obtained at the time of implant placement [17,18]. The maximum value of the insertion torque was recorded in Newton centimeter (Ncm) [16,18].

In 1996, Meredith et al. [19] developed a noninvasive clinical method to measure implant stability as RFA by using an Osstell^®^ device that can be used for multiple times both intraoperatively and during the follow-up time [19]. The resonance frequencies vary according to the different levels of implant stability, which is presented through an implant stability quotient (ISQ). To measure de ISQ value, the inserted implant is attached to a transducer (SmartPeg). The Osstell^®^ device is positioned 1 mm from the transducer and four SmartPeg points are measured (mesial, distal, buccal, and lingual/palatal). The ISQ value range from 1 to100. A value of ISQ < 60 represents low stability, ≥60 ISQ ≤ 69 represents medium stability and ISQ ≥ 70 high stability [16,18]. According to our results, there was a progressive increase in IT and ISQ over time, regardless of the technique used, SD or OD. These two independent variables indicate two different characteristics of primary stability; however, they “move” together [20,21]. These results are in line with the findings of our study, in which, overall, the higher the IT, the higher the ISQ. Another study conducted by Vale de Souza et al. [16] showed that there is a positive correlation between IT and initial ISQ (correlation: 0.457; *p* = 0.022), so that the greater the IT, the greater the initial ISQ (and vice versa). Therefore, increased IT and ISQ values are positive primary stability indicators, which can be critical for immediate loading and subsequently improving osseointegration. 

According to previous studies, the use of the OD drilling technique increases bone mineral density due to the compaction-autografting and the elastic spring-back effect, which promotes increased bone to implant contact in relation to SD technique [11,22,23]. On the other hand, the conventional drilling technique limits the initial bone–implant interaction due to the excavation of nucleated bone remnants, the amount of which can vary due to factors such as drilling speed, time and the use of irrigation in the osteotomy [15]. Buchter et al. [24] argues that the osteotome technique hinders the bone remodeling unit, causing ultrastructural microdamage, which can significantly reduce biomechanical stability shortly after implant placement [25]. Several studies showed that the osteotomized group exhibited microfractures, which was evident histologically, and the measured removal torque values were significantly lower for the same group compared to the non-condensed group. Thus, it has been concluded that traumatic damage to the bone delays the achievement of secondary stability and extends the osseointegration period to repair bone tissue microdamage, which stimulates the activation of osteoclasts [26].

The results of our study showed that there were no statistical differences between OD and SD groups in which concerns the IT and ISQ overall values, which supports the null hypothesis that the drilling technique may not influence clinical parameters of implant primary stability up to 6 months after implant placement. Although most of the studies carried out support the opposite hypothesis, it is important to consider that most of them were carried out on animals. For this reason, more human studies are needed in order to make the comparison of results as reliable as possible.

With respect to the arch, the analyses of the overall ISQ values showed an upward trend in both groups in the maxilla and mandible. According to the evidence, higher ISQ values are expected in the mandible compared to the maxilla, which is in line with our results. However, there were no statistical differences among OD and SD groups, especially between T1 and T2. This can be explained by the increased bone to implant contact that occurs during the osseointegration [15].

Despite the results obtained, in general, we can state that although IT and ISQ are two independent variables, high levels of IT also showed high ISQ values, which represents good indicators of primary stability.

In accordance with the literature, the primary stability of the implant can be significantly influenced by the macrogeometry of the implant. Some studies showed that hybrid (apical cylindrical and crestal conical) and conical designs provided the greatest primary stability [27,28]. The growing popularity of tapered implants can be attributed to their simplicity of use in clinical settings, shorter drilling sequences, and the possibility of shorter healing times and less trauma during the osteotomy. The lateral compressive forces on the cortical bone may be a significant reason for their increased primary stability [29,30]. Studies conducted on animals suggested that a larger diameter were positively correlated with greater primary stability [31,32]. Thus, a larger implant diameter improves load distribution by increasing primary stability and functional surface area. Nonetheless, a large number of studies have demonstrated that, in lower-quality bone, implants with smaller diameters can still establish adequate primary stability [32]. Our findings are in accordance with this theory, in which statistically significant differences were seen between the mean IT value and the SD technique in relation to regular implants, which showed significantly higher values when compared to Narrow implants (*p* = 0.034). The average ISQ values did not vary, but always increased over time regardless of the technique used; this could be explained by the percentage of new bone formation over time. 

An increase in IT and RFA (ISQ) values were also favorably correlated with implant length. It is well known that the use of a long, tilted implant is a method of improving the IT before immediate load rehabilitation [28]. In fact, it is directly correlated with the overall surface area in contact with the bone [28,29]. The results of the present study showed statistically significant differences in the mean IT values due to the length of the implant in the OD group (*p* = 0.041) with significantly lower mean IT values for the Regular implants compared to the Long. Regarding the ISQ, there were no differences in relation to the length of the implants considered, regardless of the technique used.

Some studies indicate that the availability of cortical and trabecular bone at the implant interface may affect the biomechanical stability of the implant and the bone healing response [4,28]. For this reason, it is important to understand the particularities, characteristics, and differences and anatomy of the maxilla and mandible. According to the Lekholm and Zarb (1985) classification (the most popular classification of bone quality), bone types are classified based on the amount of cortical versus trabecular bone from I to IV [33,34,35]. The biomechanical properties of osteoporotic bone are similar to those of type IV bone, and do not provide appropriate stability for implants. Another important aspect is bone density according to anatomical location which is characterized by the Norton and Gamble classification. Norton and Gamble described different bone density range according to their typical anatomical locations in the maxilla and mandible. All of the subjectively rated areas in each of the four qualities were subsequently grouped together so that a range of Houndsfield (HU) values could be assigned to each specific quality [36]. Low-density bone (type III and type IV), commonly seen in the posterior mandible, especially in elderly patients, represents a high percentage of those seeking implant treatment.

The results of the present study showed that there were statistical differences in relation to the arch and the type of osteotomy with respect to IT. IT and ISQ were higher in the mandible than in the maxilla for both the SD and OD techniques. These results are in line with the study by Turkyilmaz et al. [37], which found a strong relationship between bone density and ISQ values.

With regard to area, in general, the anterior region showed higher IT values compared to the posterior area for both techniques. These results can be explained by the bone density in the anterior region compared to the posterior region of the arch. However, in terms of technique, the anterior region of the OD group showed higher IT values compared to the SD. These results are in compliance with the study by Bergamo et.al. [23], with 150 implants, in which the anterior region showed increased IT in the OD group when compared to the SD group.

Although this was not the aim of the present study, in clinical practice, achieving high levels of biomechanical stability has become more necessary to support the current tendency toward early loading protocols. In a study by Trisi et al. [38], immediate loading can be performed when IT value is at least 45 Ncm and ISQ at least 68. Thus, according to the results, rehabilitation with immediate loading was a possible option for implants with an ISQ > 68, which can be especially useful for the posterior maxillary region, which has low-density bone that makes immediate loading protocols difficult.

A larger sample of wide implants would be necessary in order to understand whether there was a change in primary stability parameters between osseodensification and subtractive conventional drilling. Furthermore, more human studies are needed, especially on low-density bone (type III and IV), so that the results can be compared as reliably as possible. Most studies on this technique have been carried out on animals and not humans, which makes it difficult to compare the results.

## 5. Conclusions

The results strongly indicate that OD does not have a negative influence on osseointegration compared to conventional subtractive osteotomy. Furthermore, the tapered implant design may compensate for the low stability expected in soft bone, and dense bone may compensate for short implant length if required by the anatomical bone conditions.

Osseodensification appears to be a viable method for increasing bone quantity and quality, but the literature’s results are inconclusive and should be read thoughtfully.

## Figures and Tables

**Table 1:** Conventional drilling sequence for Ø 3.3 mm BLT implant.

Straumann^®^ BLT		Drilling Sequence					
Geometry	Implant Diameter	Type of Bone	Needle Drill Ø 1.6 mm (800 rpm)	Pilot Drill Ø 2.2 mm (800 rpm)	BLT Drill Ø 2.8 mm (600 rpm)	Profile Drill Ø 3.3 mm (300 rpm)	BLT Tap Ø 3.3 mm (15 rpm)
Tapered	Ø 3.3 mm	Type I	•	•	•	•	• *
		Type II	•	•	•	• *	
		Type III	•	•	• *		
		Type IV	•	• *			

• Performing the osteotomy; • * Osteotomy and implant placement.

**Table 2:** Conventional drilling sequence for Ø 4.1 mm BLT implant.

Straumann^®^ BLT		Drilling Sequence						
Geometry	Implant Diameter	Type of Bone	Needle Drill Ø 1.6 mm (800 rpm)	Pilot Drill Ø 2.2 mm (800 rpm)	BLT Drill Ø 2.8 mm (600 rpm)	BLT Drill Ø 3.5 mm (500 rpm)	Profile Drill Ø 4.1 mm (300 rpm)	BLT Tap Ø 4.1 mm (15 rpm)
Tapered	Ø 4.1 mm	Type I	•	•	•	•	•	• *
		Type II	•	•	•	•	• *	
		Type III	•	•	•	• *		
		Type IV	•	•	•	• *		

• Performing the osteotomy; • * Osteotomy and implant placement.

**Table 3:** Conventional drilling sequence for Ø 4.8 mm BLT implant.

Straumann^®^ BLT		Drilling Sequence							
Geometry	Implant Diameter	Type of Bone	Needle Drill Ø 1.6 mm (800 rpm)	Pilot Drill Ø 2.2 mm (800 rpm)	BLT Drill Ø 2.8 mm (600 rpm)	BLT Drill Ø 3.5 mm (500 rpm)	BLT Drill Ø 4.2 mm (400 rpm)	Profile Drill Ø 4.8 mm (300 rpm)	BLT Tap Ø 4.8 mm (15 rpm)
Tapered	Ø 4.8 mm	Type I	•	•	•	•	•	•	• *
		Type II	•	•	•	•	•	• *	
		Type III	•	•	•	•	• *		
		Type IV	•	•	•	• *			

• Performing the osteotomy; • * Osteotomy and implant placement.

**Table 4:** Drilling sequence of Densah^®^ Burs.

Straumann^®^		Soft Bone (Type III and IV)			
Geometry	Implant Diameter	Pilot	Bur 1	Bur 2	Bur 3
Tapered	Ø 3.3 mm	Pilot drill	VT1828 * (2.3)	—	—
	Ø 4.1 mm	Pilot drill	VT1525 (2.0)	VT2535 * (3.0)	—
	Ø 4.8 mm	Pilot drill	VT1525 (2.0)	VT2535 (3.0)	VT3545 * (4.0)

* Implant placement.

**Table 5:** Distribution of members by gender.

Gender	n	%	CI 95.0%	
			LCL	UCL
F	55	61.1%	50.8%	70.7%
M	35	38.9%	29.3%	49.2%
Total	90	100.0%		

Note: F—female; M—male; n—number of subjects and percentages; LCL—Lower Control Limit; UCL—Upper Control Limit.

**Table 6:** Summary statistics for age by gender.

		Gender		Total
		F	M	
Age	Mean	47.7	50.3	48.7
	Median	49.0	48.0	48.5
	Standard deviation	12.7	11.2	12.1
	Minimum	19.0	20.0	19.0
	Maximum	72.0	69.0	72.0
	Percentile 25	37.0	44.0	42.0
	Percentile 75	56.0	59.0	57.0

Note: F—female; M—male.

**Table 7:** Summary statistics for the individual’s characteristics.

		n	%	CI 95.0%	
				LCL	UCL
Smoker	N	74	82.2%	73.4%	89.0%
	Y	16	17.8%	11.0%	26.6%
	Total	90	100.0%		
Systemic Disease	N	71	78.9%	69.6%	86.3%
	Y	19	21.1%	13.7%	30.4%
	Total	90	100.0%		
Number of cigarettes/day	4	1	6.3%	0.7%	25.7%
	5	1	6.3%	0.7%	25.7%
	6	1	6.3%	0.7%	25.7%
	10	5	31.3%	13.1%	55.6%
	15	2	12.5%	2.7%	34.4%
	20	6	37.5%	17.4%	61.7%
	Total	16	100.0%		

Note: N—no; n—number of subjects and percentages; LCL—Lower Control Limit; UCL—Upper Control Limit; Y—yes.

**Table 8:** Implant-related characterization.

		Surgical Techniques											
		SD				OD				Total			
		n	%	CI 95.0%		n	%	CI 95.0%		n	%	CI 95.0%	
				LCL for %	UCL for %			LCL for %	UCL for %			LCL for %	UCL for %
Implant diameter (mm)	Narrow	26	16.3%	11.2%	22.5%	25	21.2%	14.6%	29.2%	51	18.3%	14.1%	23.2%
	Regular	132	82.4%	76.1%	87.8%	90	76.3%	68.0%	83.2%	222	79.9%	74.8%	84.2%
	Wide	2	1.3%	0.3%	3.9%	3	2.5%	0.7%	6.6%	5	1.8%	0.7%	3.9%
	Total	160	100%	.	.	118	100.0%	.	.	278	100.0%	.	.
Implant length (mm)	8	33	20.6%	14.9%	27.4%	30	25.4%	18.2%	33.8%	63	22.7%	18.0%	27.8%
	10	65	40.6%	33.2%	48.3%	51	43.2%	34.5%	52.2%	116	41.7%	36.0%	47.6%
	12	37	23.1%	17.1%	30.1%	25	21.2%	14.6%	29.2%	62	22.3%	17.7%	27.5%
	14	9	5.6%	2.8%	10.0%	7	5.9%	2.7%	11.3%	16	5.8%	3.5%	9.0%
	16	7	4.4%	2.0%	8.4%	3	2.5%	0.7%	6.6%	10	3.6%	1.9%	6.3%
	18	9	5.6%	2.8%	10.0%	2	1.7%	0.4%	5.3%	11	4.0%	2.1%	6.7%
	Total	160	100.0%	.	.	118	100.0%	.	.	278	100.0%	.	.
Arch	Maxilla	78	48.8%	41.1%	56.5%	86	72.9%	64.4%	80.3%	164	59.0%	53.1%	64.7%
	Mandible	82	51.3%	43.5%	58.9%	32	27.1%	19.7%	35.6%	114	41.0%	35.3%	46.9%
	Total	160	100.0%	.	.	118	100.0%	.	.	278	100.0%	.	.
Position	11	1	0.6%	0.1%	2.9%	0	0.0%	.	.	1	0.4%	0.0%	1.7%
	12	3	1.9%	0.5%	4.9%	2	1.7%	0.4%	5.3%	5	1.8%	0.7%	3.9%
	13	4	2.5%	0.8%	5.8%	2	1.7%	0.4%	5.3%	6	2.2%	0.9%	4.4%
	14	15	9.4%	5.6%	14.6%	6	5.1%	2.1%	10.2%	21	7.6%	4.9%	11.1%
	15	15	9.4%	5.6%	14.6%	13	11.0%	6.3%	17.6%	28	10.1%	6.9%	14.0%
	16	9	5.6%	2.8%	10.0%	14	11.9%	7.0%	18.6%	23	8.3%	5.5%	11.9%
	17	3	1.9%	0.5%	4.9%	3	2.5%	0.7%	6.6%	6	2.2%	0.9%	4.4%
	21	0	0.0%	.	.	1	0.8%	0.1%	3.9%	1	0.4%	0.0%	1.7%
	22	5	3.1%	1.2%	6.7%	2	1.7%	0.4%	5.3%	7	2.5%	1.1%	4.9%
	23	2	1.3%	0.3%	3.9%	2	1.7%	0.4%	5.3%	4	1.4%	0.5%	3.4%
	24	5	3.1%	1.2%	6.7%	12	10.2%	5.7%	16.6%	17	6.1%	3.7%	9.4%
	25	11	6.9%	3.7%	11.6%	11	9.3%	5.1%	15.6%	22	7.9%	5.2%	11.5%
	26	5	3.1%	1.2%	6.7%	13	11.0%	6.3%	17.6%	18	6.5%	4.0%	9.8%
	27	0	0.0%	.	.	5	4.2%	1.6%	9.0%	5	1.8%	0.7%	3.9%
	32	1	0.6%	0.1%	2.9%	2	1.7%	0.4%	5.3%	3	1.1%	0.3%	2.9%
	34	6	3.8%	1.6%	7.6%	1	0.8%	0.1%	3.9%	7	2.5%	1.1%	4.9%
	35	3	1.9%	0.5%	4.9%	2	1.7%	0.4%	5.3%	5	1.8%	0.7%	3.9%
	36	22	13.8%	9.1%	19.7%	4	3.4%	1.2%	7.9%	26	9.4%	6.3%	13.2%
	37	8	5.0%	2.4%	9.2%	3	2.5%	0.7%	6.6%	11	4.0%	2.1%	6.7%
	42	3	1.9%	0.5%	4.9%	0	0.0%	.	.	3	1.1%	0.3%	2.9%
	44	5	3.1%	1.2%	6.7%	2	1.7%	0.4%	5.3%	7	2.5%	1.1%	4.9%
	45	7	4.4%	2.0%	8.4%	7	5.9%	2.7%	11.3%	14	5.0%	2.9%	8.1%
	46	17	10.6%	6.6%	16.1%	9	7.6%	3.8%	13.5%	26	9.4%	6.3%	13.2%
	47	10	6.3%	3.3%	10.8%	2	1.7%	0.4%	5.3%	12	4.3%	2.4%	7.2%
	Total	160	100.0%	.	.	118	100.0%	.	.	278	100.0%	.	.
Operated Area	Anterior	19	11.9%	7.6%	17.6%	11	9.3%	5.1%	15.6%	30	10.8%	7.6%	14.8%
	Posterior	141	88.1%	82.4%	92.4%	107	90.7%	84.4%	94.9%	248	89.2%	85.2%	92.4%
	Total	160	100.0%	.	.	118	100.0%	.	.	278	100.0%	.	.

Note: n—number of implants and percentages; LCL—Lower Control Limit; OD—osseodensification; SD—subtractive conventional drilling; UCL—Upper Control Limit.

**Table 9:** Pearson correlation between the IT and ISQ T1 values and the variables under study.

	Maxilla ISQ T1	Mandible ISQ T1	Anterior ISQ T1	Posterior ISQ T1	Narrow ISQ T1	Regular ISQ T1	Wide ISQ T1	Short ISQ T1	Regular ISQ T1	Long ISQ T1	SD ISQ T1	OD ISQ T1	Total ISQ
Maxilla IT	r = 0.192 * p = 0.014												
Mandible IT		r = 0.315 * p < 0.001											
Anterior IT			r = −0.003 *p* = 0.988										
Posterior IT				r = 0.326 * p < 0.001									
Narrow IT					r = 0.195 *p* = 0.171								
Regular IT						r = 0.242 * p < 0.001							
Wide IT							r = 0.903 * p = 0.036						
Short IT								r = 0.413 * p < 0.001					
Regular IT									r = 0.310 * p < 0.001				
Long IT										r = 0.058 *p* = 0.734			
SD IT											r = 0.290 * p < 0.001		
OD IT												r = 0.221 * p = 0.016	
Total IT													r = 0.263 * p < 0.001

Note: ISQ T1—implant stability quotient in the surgical phase of implant placement; IT—insertion torque; OD—ossedensification; *p* = level of significance; SD—subtractive conventional drilling. * significant for the 5% decision rule used.

## Data Availability

The data can be accessed by contacting the corresponding author.

## References

[1] Branemark P., Adell R., Albrektsson T., Lundkvist U., Rockler B. (1983). Osseointegrated titanium fixtures in the treatment of edentulousness. Biomaterials.

[2] Witek L., Neiva R., Alifarag A., Shahraki F., Sayah G., Tovar N., Lopez C.D., Gil L., Coelho P.G. (2019). Absence of Healing Impairment in Osteotomies Prepared via Osseodensification Drilling. Int. J. Periodontics Restor. Dent..

[3] Jarikian S., Jaafo M.H., Al-Nerabieah Z. (2021). Clinical evaluation of two techniques for narrow alveolar ridge expansion: Clinical study. Int. J. Dent. Oral Sci..

[4] de Oliveira P.G.F.P., Bergamo E.T.P., Neiva R., Bonfante E.A., Witek L., Tovar N., Coelho P.G. (2018). Osseodensification outperforms conventional implant subtractive instrumentation: A study in sheep. Mater. Sci. Eng. C.

[5] Ottoni J.M., Oliveira Z.F., Mansini R., Cabral A.M. (2005). Correlation between placement torque and survival of single-tooth implants. Int. J. Oral Maxillofac. Implant..

[6] Trisi P., Todisco M., Consolo U., Travaglini D. (2011). High versus low implant insertion torque: A histologic, histomorphometric, and biomechanical study in the sheep mandible. Int. J. Oral Maxillofac. Implant..

[7] Inchingolo A.D., Inchingolo A.M., Bordea I.R., Xhajanka E., Romeo D.M., Romeo M., Zappone C.M.F., Malcangi G., Scarano A., Lorusso F. (2021). The effectiveness of osseodensification drilling protocol for implant site osteotomy: A systematic review of the literature and meta-analysis. Materials.

[8] Bonfante E.A., Jimbo R., Witek L., Tovar N., Neiva R., Torroni A., Coelho P.G. (2019). Biomaterial and biomechanical considerations to prevent risks in implant therapy. Periodontology 2000.

[9] Formiga M.d.C., Grzech-Lesniak K., Moraschini V., Shibli J.A., Neiva R. (2022). Effects of Osseodensification on Immediate Implant Placement: Retrospective Analysis of 211 Implants. Materials.

[10] Trisi P., Berardini M., Falco A., Podaliri Vulpiani M. (2016). New osseodensification implant site preparation method to increase bone density in low-density bone: In vivo evaluation in sheep. Implant Dent..

[11] Lahens B., Neiva R., Tovar N., Alifarag A.M., Jimbo R., Bonfante E.A., Bowers M.M., Cuppini M., Freitas H., Witek L. (2016). Biomechanical and histologic basis of osseodensification drilling for endosteal implant placement in low density bone. An experimental study in sheep. J. Mech. Behav. Biomed. Mater..

[12] Koutouzis T., Huwais S., Hasan F., Trahan W., Waldrop T., Neiva R. (2019). Alveolar Ridge Expansion by Osseodensification-Mediated Plastic Deformation and Compaction Autografting: A Multicenter Retrospective Study. Implant Dent..

[13] Ann D., Hofbauer M., Huwais S. (2018). Osseodensification Facilitates Ridge Expansion 2nd Case Study. Implant. Pract..

[14] Dwan K., Li T., Altman D.G., Elbourne D. (2019). CONSORT 2010 statement: Extension to randomised crossover trials. BMJ.

[15] Alifarag A.M., Lopez C.D., Neiva R.F., Tovar N., Witek L., Coelho P.G. (2018). Atemporal osseointegration: Early biomechanical stability through osseodensification. J. Orthop. Res..

[16] Do Vale Souza J.P., De Moraes Melo Neto C.L., Piacenza L.T., Freitas Da Silva E.V., De Melo Moreno A.L., Penitente P.A., Brunetto J.L., Dos Santos D.M., Goiato M.C. (2021). Relation between Insertion Torque and Implant Stability Quotient: A Clinical Study. Eur. J. Dent..

[17] Sarfaraz H., Johri S., Sucheta P., Rao S. (2018). Study to assess the relationship between insertion torque value and implant stability quotient and its influence on timing of functional implant loading. J. Indian. Prosthodont. Soc..

[18] Stoilov M., Shafaghi R., Stark H., Marder M., Kraus D., Enkling N. (2023). Influence of Implant Macro-Design, -Length, and -Diameter on Primary Implant Stability Depending on Different Bone Qualities Using Standard Drilling Protocols—An In Vitro Analysis. J. Funct. Biomater..

[19] Meredith N., Alleyne D., Cawley P. (1996). Quantitative determination of the stability of the implant-tissue interface using resonance frequency analysis. Clin. Oral Implant. Res..

[20] Filho L.C.M., Cirano F.R., Hayashi F., Feng H.S., Conte A., Dib L.L., Casati M.Z. (2014). Assessment of the correlation between insertion torque and resonance frequency analysis of implants placed in bone tissue of different densities. J. Oral Implantol..

[21] Makary C., Rebaudi A., Sammartino G., Naaman N. (2012). Implant primary stability determined by resonance frequency analysis: Correlation with insertion torque, histologic bone volume, and torsional stability at 6 weeks. Implant Dent..

[22] Fontes Pereira J., Costa R., Nunes Vasques M., Salazar F., Mendes J.M., Infante da Câmara M. (2023). Osseodensification: An Alternative to Conventional Osteotomy in Implant Site Preparation: A Systematic Review. J. Clin. Med..

[23] Bergamo E.T.P., Zahoui A., Barrera R.B., Huwais S., Coelho P.G., Karateew E.D., Bonfante E.A. (2021). Osseodensification effect on implants primary and secondary stability: Multicenter controlled clinical trial. Clin. Implant Dent. Relat. Res..

[24] Büchter A., Kleinheinz J., Wiesmann H.P., Kersken J., Nienkemper M., Von Weyhrother H., Joos U., Meyer U. (2005). Biological and biomechanical evaluation of bone remodelling and implant stability after using an osteotome technique. Clin. Oral Implant. Res..

[25] Zitzmann N.U., Schärer P. (1998). Sinus elevation procedures in the resorbed posterior maxilla: Comparison of the crestal and lateral approaches. Oral Surg. Oral Med. Oral Pathol. Oral Radiol. Endod..

[26] Frost H.M. (1998). A brief review for orthopedic surgeons: Fatigue damage (microdamage) in bone (its determinants and clinical implications). J. Orthop. Sci..

[27] Heimes D., Becker P., Pabst A., Smeets R., Kraus A., Hartmann A., Sagheb K., Kämmerer P.W. (2023). How does dental implant macrogeometry affect primary implant stability? A narrative review. Int. J. Implant Dent..

[28] Raz P., Meir H., Levartovsky S., Sebaoun A., Beitlitum I. (2022). Primary Implant Stability Analysis of Different Dental Implant Connections and Designs—An In Vitro Comparative Study. Materials.

[29] Costa J.A., Mendes J.M., Salazar F., Pacheco J.J., Rompante P., Moreira J.F., Mesquita J.D., Adubeiro N., da Câmara M.I. (2024). Osseodensification vs. Conventional Osteotomy: A Case Series with Cone Beam Computed Tomography. J. Clin. Med..

[30] Yamaguchi Y., Shiota M., Munakata M., Kasugai S., Ozeki M. (2015). Effect of implant design on primary stability using torque-time curves in artificial bone. Int. J. Implant Dent..

[31] Al-Nawas B., Groetz K.A., Goetz H., Duschner H., Wagner W. (2008). Comparative histomorphometry and resonance frequency analysis of implants with moderately rough surfaces in a loaded animal model. Clin. Oral Implant. Res..

[32] Ivanoff C.J., Sennerby L., Johansson C., Rangert B., Lekholm U. (1997). Influence of implant diameters on the integration of screw implants: An experimental study in rabbits. Int. J. Oral Maxillofac. Surg..

[33] Kido H., Schulz E.E., Kumar A., Lozada J., Saha S. (1997). Implant diameter and bone density: Effect on initial stability and pull-out resistance. J. Oral Implantol..

[34] Alghamdi H.S. (2018). Methods to improve osseointegration of dental implants in low quality (type-IV) bone: An overview. J. Funct. Biomater..

[35] Lekholm U., Zarb G., Brånemark P.I., Zarb G.A., Albrektsson T. (1985). Patient selection and preparation. Tissue-Integrated Prostheses: Osseointegration in Clinical Dentistry.

[36] Norton M.R., Gamble C. (2001). Bone classification: An objective scale of bone density using the computerized tomography scan. Clin. Oral Implant. Res..

[37] Turkyilmaz I., Tumer C., Ozbek E.N., Tözüm T.F. (2007). Relations between the bone density values from computerized tomography, and implant stability parameters: A clinical study of 230 regular platform implants. J. Clin. Periodontol..

[38] Trisi P., Perfetti G., Baldoni E., Berardi D., Colagiovanni M., Scogna G. (2009). Implant micromotion is related to peak insertion torque and bone density. Clin. Oral Implant. Res..

